# Factors associated with the continuum of care of HIV-infected patients in Belgium

**DOI:** 10.7448/IAS.17.4.19534

**Published:** 2014-11-02

**Authors:** Dominique Van Beckhoven, Patrick Lacor, Michel Moutschen, Denis Piérard, André Sasse, Dolorès Vaira, Sigi Van den Wijngaert, Bernard Vandercam, Marc Van Ranst, Eric Van Wijngaerden, Linos Vandekerckhove, Chris Verhofstede, Ruth Verbrugge, Rémy Demeester, Stéphane De Wit, Eric Florence, Katrien Fransen, Marie-Luce Delforge, Jean-Christophe Goffard, Patrick Goubau, BREACH

**Affiliations:** 1 Epidemiology of Infectious Diseases Unit, Scientific Institute of Public Health, Brussels, Belgium; 2 AIDS Reference Center, Universitair Ziekenhuis Brussel, Brussels, Belgium; 3 AIDS Reference Center, CHU de Liège, Liege, Belgium; 4 AIDS Reference Laboratory, Universitair Ziekenhuis Brussel, Brussels, Belgium; 5 AIDS Reference Laboratory, Liège University, Liege, Belgium; 6 Laboratory of Microbiology, CHU Saint-Pierre, Brussels, Belgium; 7 AIDS Reference Center, Cliniques Universitaires Saint-Luc, Brussels, Belgium; 8 AIDS Reference Laboratory, KU Leuven, Leuven, Belgium; 9 AIDS Reference Center, UZ Leuven, Leuven, Belgium; 10 UZ Gent, AIDS Reference Center, Ghent, Belgium; 11 UZ Gent, AIDS Reference Laboratory, Ghent, Belgium; 12 AIDS Reference Center, CHU de Charleroi, Charleroi, Belgium; 13 AIDS Reference Center, CHU Saint-Pierre, Brussels, Belgium; 14 AIDS Reference Center, Instituut Tropische Geneeskunde, Antwerp, Belgium; 15 AIDS Reference Laboratory, Instituut Tropische Geneeskunde, Antwerp, Belgium; 16 AIDS Reference Laboratory, University Hospital ULB Erasme, Brussels, Belgium; 17 AIDS Reference Center, University Hospital ULB Erasme, Brussels, Belgium; 18 AIDS Reference Laboratory, Université Catholique de Louvain, Brussels, Belgium; 19 Belgian Research on AIDS and HIV Consortium, Belgium

## Abstract

**Introduction:**

We studied factors associated with the continuum of HIV care in Belgium.

**Methods:**

Data of the national registration of new HIV diagnosis and of the national cohort of HIV-infected patients in care were combined to obtain estimates of and factors related with proportions of HIV-infected patients in each step of the continuum of care from diagnosis to suppressed viral load (VL). Factors associated with ignorance of HIV seropositivity were analyzed among patients co-infected with HIV and STI in the Belgian STI sentinel surveillance network. Associated factors were identified by multivariate logistic regression.

**Results:**

Among 4038 individuals diagnosed with HIV between 2007 and 2010, 90.3% were linked to care. Of 11684 patients in care in 2010, 90.8% were retained in care up to the following year, 88.3% of those were on ART, of whom 95.3% had suppressed VL (<500 cp/ml) (Figure 1). In multivariate analyses, factors associated with ignoring HIV+ status were being younger (p<0.001), being heterosexual compared to MSM, and of a region of origin other than Belgium, Sub-Saharan Africa and Europe. Non-Belgian regions of origin were associated with lower entry and retention in care (p<0.001 for both). Preoperative HIV testing was associated with lower entry in care (p=0.003). MSM had a higher retention in care (p<0.001), whilst IDU had lower retention (p=0.004). Low CD4 at first clinical contact and clinical reasons for HIV testing were independently associated with being on ART (p<0.001 for both); whilst prenatal HIV diagnosis was associated with lower proportion on ART (p=0.016) and lower proportion with suppressed VL among those on ART (p=0.005). Older age was associated with both being on ART and having suppressed VL among those on ART (p=0.007 and p<0.001 respectively), independently of time since HIV diagnosis (Table 1).

**Conclusions:**

Regions of origin and risk groups (MSM/heterosexual/IDU) are the main factors associated with ignorance of HIV seropositivity, entry and retention in care, but once the HIV patient is retained in care, no effect of these factors on the proportions on ART and with suppressed VL are observed. The association of prenatal HIV diagnosis and proportions on ART and with suppressed VL could be biased by transitory CD4 disturbances during pregnancy and ART discontinuation after pregnancy. The higher probabilities of older patients to be on ART and have suppressed VL once retained in care could be influenced by factors not studied here like comorbidities, adherence or duration on ART.

**Figure 1:**
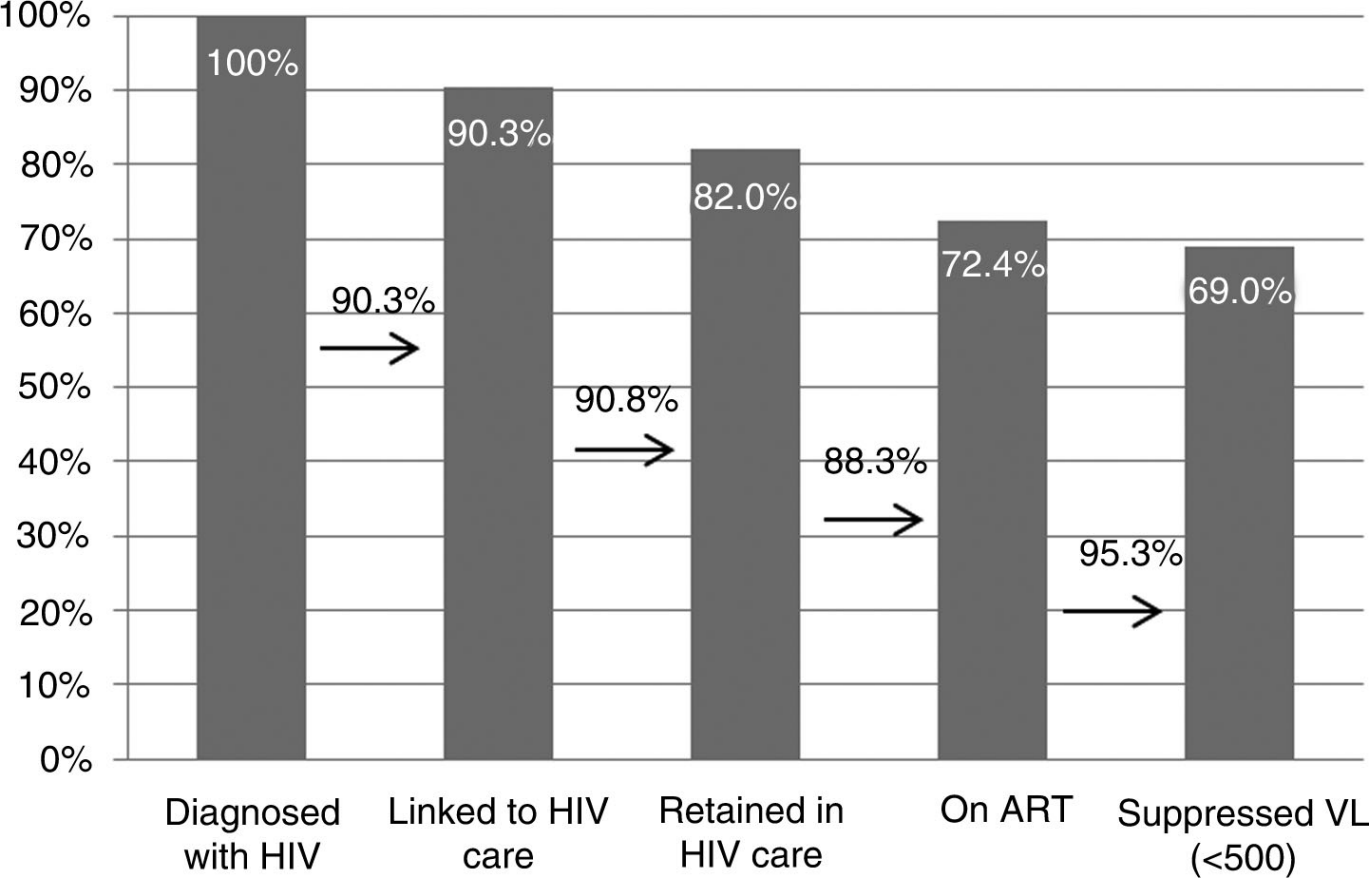
The continuum of HIV care in Belgium.

**Table 1:** Adjusted OR for factors associated with each step of the continuum of HIV care

Risk factors	Adjusted OR (95% CI)^a^ Undiagnosed HIV +	Adjusted OR (95% CI)^a^ No entry in care	Adjusted OR (95% CI)^a^ No retention	Adjusted OR (95% CI)^a^ On ART	Adjusted OR (95% CI)^a^ Suppressed VL (<500 cp/ml)
Sex					
Male	1	1	1	1	1
Female	1.32 (0.39–4.44)	0.88 (0.58–1.34)	0.87 (0.70–1.07)	0.76 (0.55–1.04)^b^	1.02 (0.73–1.42)
Age at diagnosis					
<40 yrs	1	1	1	1	1
≥40 yrs	0.42 (0.27–0.64)	0.98 (0.69–1.39)	1.05 (0.86–1.28)	1.31 (1.04–1.65)^b^	1.75 (1.27–2.42)
Way of transmission					
Heterosexual	1	1	1	1	1
MSM	0.39 (0.16–0.95)	0.86 (0.52–1.44)	0.61 (0.47–0.78)	0.80 (0.58–1.09)^b^	1.06 (0.72–1.56)
IDU	/	1.50 (0.55–4.11)	1.88 (1.22–2.88)	2.42 (0.69–8.46)^b^	1.46 (0.52–4.11)
Region of origin					
Belgium	1	1	1	1	1
Sub-Saharan Africa	1.02 (0.33–3.14)	3.11 (1.84–5.26)	1.41 (1.12–1.78)	0.90 (0.66–1.23)^b^	0.73 (0.51–1.03)^b^
Europe	0.83 (0.41–1.69)	2.74 (1.59–4.71)	1.86 (1.38–2.52)	0.97 (0.67–1.40)^b^	0.97 (0.57–1.66)^b^
Other	2.25 (1.26–4.04)	3.23 (1.79–5.83)	1.54 (1.10–2.17)	0.90 (0.59–1.37)^b^	0.89 (0.51–1.48)
Reason for testing					
Patient's request	/	1	1	1	1
Clinical arguments	/	0.98 (0.64–1.50)	0.95 (0.74–1.21)	1.76 (1.34–2.31)^b^	0.92 (0.63–1.35)
Prenatal	/	1.14 (0.51–2.52)	1.16 (0.75–1.78)	0.49 (0.30–0.81)^b^	0.42 (0.23–0.78)
Preoperative	/	3.22 (1.50–6.89)	1.21 (0.72–2.04)	1.14 (0.56–2.31)^b^	0.85 (0.35–2.04)
Other	/	0.92 (0.55–1.54)	1.13 (0.86–1.49)	1.54 (1.11–2.14)^b^	1.03 (0.65–1.63)
CD4 at first visit					
CD4 ≥350	/	/	1	1	1
CD4 <350	/	/	1.16 (0.89–1.51)	8.02 (5.80–11.11)	1.16 (0.78–1.70)

Note: Rem: p<0.05, statistically significant variables presented in italic.

a Adjusted for sex, age at diagnosis, nationality and ay of transmission

b additionally adjusted for CD4 value at first visit.

